# Association of serum zinc with mineral stress in chronic kidney disease

**DOI:** 10.1093/ckj/sfae258

**Published:** 2024-08-20

**Authors:** Azmat Sohail, Jakob Obereigner, Gregor Mitter, Thomas Schmid, Anna-Sofie Hofer, Gerhard Schuster, Astrid Hügl, Angelika H Dorninger, Markus Mandl, Andreas Pasch, Helmut K Lackner, Ilona Papousek, Benjamin Dieplinger, Susanne Suessner, Marlies Antlanger, Daniel Cejka, Ioana Alesutan, Jakob Voelkl

**Affiliations:** Institute for Physiology and Pathophysiology, Johannes Kepler University Linz, Linz, Austria; Institute for Physiology and Pathophysiology, Johannes Kepler University Linz, Linz, Austria; Institute for Physiology and Pathophysiology, Johannes Kepler University Linz, Linz, Austria; AMD GmbH, Linz, Austria; Department of Medicine III - Nephrology, Hypertension, Transplantation Medicine, Rheumatology, Geriatrics, Ordensklinikum Linz, Linz, Austria; Red Cross Transfusion Service of Upper Austria, Austrian Red Cross, Linz, Austria; Red Cross Transfusion Service of Upper Austria, Austrian Red Cross, Linz, Austria; Red Cross Transfusion Service of Upper Austria, Austrian Red Cross, Linz, Austria; Institute for Physiology and Pathophysiology, Johannes Kepler University Linz, Linz, Austria; Institute for Physiology and Pathophysiology, Johannes Kepler University Linz, Linz, Austria; Calciscon AG, Biel, Switzerland; Division of Physiology and Pathophysiology, Otto Loewi Research Center, Medical University of Graz, Graz, Austria; Institute of Psychology, Biological Psychology Unit, University of Graz, Graz, Austria; Department of Laboratory Medicine, Konventhospital Barmherzige Brueder Linz and Ordensklinikum Linz, Linz, Austria; Red Cross Transfusion Service of Upper Austria, Austrian Red Cross, Linz, Austria; Department of Internal Medicine 2, Kepler University Hospital and Johannes Kepler University, Linz, Austria; Department of Medicine III - Nephrology, Hypertension, Transplantation Medicine, Rheumatology, Geriatrics, Ordensklinikum Linz, Linz, Austria; Institute for Physiology and Pathophysiology, Johannes Kepler University Linz, Linz, Austria; Institute for Physiology and Pathophysiology, Johannes Kepler University Linz, Linz, Austria; Department of Nephrology and Medical Intensive Care, Charité-Universitätsmedizin Berlin, Corporate Member of Freie Universität Berlin and Humboldt Universität zu Berlin, Berlin, Germany; DZHK (German Centre for Cardiovascular Research), Partner Site Berlin, Berlin, Germany

**Keywords:** calciprotein particles, chronic kidney disease, mineral stress, serum calcification propensity, zinc

## Abstract

**Background:**

The excessive cardiovascular mortality of patients with chronic kidney disease (CKD) could be linked to mineral stress, the biological consequence of calcium-phosphate nanoparticle exposure. This study investigated whether zinc is associated with mineral stress markers in CKD.

**Methods:**

*Z*inc and T50 (serum calcification propensity) as well as hydrodynamic radius of secondary calciprotein particles (CPP2) were measured in blood donors and CKD patients with/out dialysis.

**Results:**

Serum zinc concentrations and T50 were reduced, while CPP2 radius was increased in CKD patients. Serum zinc levels positively correlated with T50 and inversely correlated with CPP2 radius. In a hierarchical linear regression model, T50 was associated with age, calcium, phosphate, magnesium and albumin. Addition of zinc significantly improved prediction of the model, confirming an additional contribution of zinc to T50. Similar observations were made for the association of zinc and CPP2 radius, but spiking experiments indicated that zinc may stronger modify T50 than CPP2 radius. Also, urinary zinc excretion was increased in patients with kidney disease and correlated to T50 and CPP2 radius. *S*erum zinc further correlated with markers of arterial stiffness in blood donors and CKD patients, but these associations did not remain significant in a multivariate linear regression model.

**Conclusions:**

Reduced serum zinc levels in CKD appear directly linked to lower T50 and associated with larger CPP2 radius. Further studies on the associations of zinc and mineral stress as well as putative therapeutic benefits of zinc supplementation are required.

## Graphical Abstract

**Graphical Abstract:**
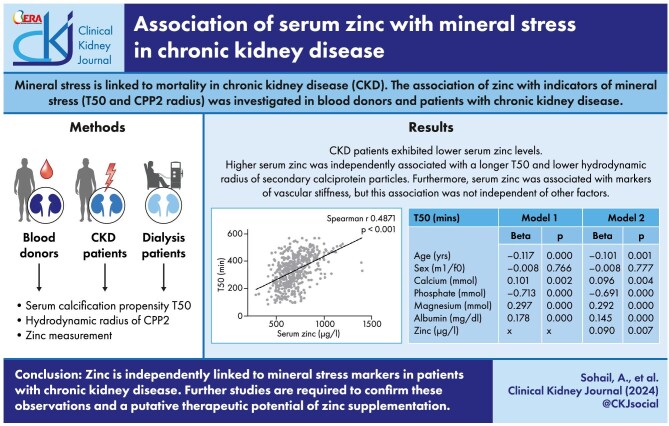

KEY LEARNING POINTS **What was known:**

- Mineral stress occurs due to chronic exposure of tissues to calciprotein particles (CPPs).
- Mineral stress markers are increased and linked to mortality in chronic kidney disease (CKD).
- This study investigated serum zinc as potential factor in the development of mineral stress in blood donors and patients with CKD.

**This study adds:**

- CKD patients exhibited lower serum zinc levels and indications of elevated mineral stress. Higher serum zinc was independently associated with a longer T50 (serum calcification propensity) and lower hydrodynamic radius of secondary CPPs.
- Furthermore, serum zinc was associated with markers of vascular stiffness, but this association was not independent of other factors.

**Potential impact:**

- These observations identify an association of zinc and mineral stress markers in patients with CKD.
- Further studies are required to confirm these observations and a putative therapeutic potential of zinc supplementation.

## INTRODUCTION

Chronic kidney disease (CKD) dramatically increases the risk for cardiovascular events and mortality [1]. A significant contribution to the increased cardiovascular risk in CKD could be due

to derangements in mineral homeostasis, especially hyperphosphatemia. Elevated phosphate levels may foster inflammation and vascular calcification [1].

The pro-inflammatory effect of hyperphosphatemia may involve mineral stress, the biological consequence of increased calcium-phosphate nanoparticle formation [2]. Human blood can be considered supersaturated with regard to calcium and phosphate, and mineralization inhibitors are required to prevent formation of larger calcium-phosphate crystals [1]. Several calcification inhibitors are known, such as pyrophosphate or the vitamin K–dependent matrix GLA protein, and their deficiency favours vascular calcification [3]. An especially important role in this system is attributed to Fetuin-A, which binds calcium-phosphate complexes to form calciprotein monomers [4]. These monomers can aggregate to form primary calciprotein particles (CPPs). When primary CPPs are not rapidly removed from the circulation, they could mature into larger secondary CPPs (CPP2) [4]. CPPs can trigger inflammatory pathways [5], linking secondary CPPs to endothelial dysfunction [5] and vascular calcification [6].

Thus, the balance between phosphate and mineralization inhibitors might be decisive for cardiovascular outcomes, especially in vulnerable groups like CKD patients. This system has been approached diagnostically by the serum calcification propensity or T50 test, which determines the half-maximal transition time of primary to secondary CPPs in individual serum samples [2]. This functional test reflects the sum of pro- and anti-calcific factors in the blood. The T50 time is reduced (reflecting higher calcification propensity) in CKD patients [7, 8]. Most importantly, a shorter T50 is associated with an increased mortality in CKD patients [7] and the general population [9]. Beyond the T50, another marker of mineral stress is CPP2 hydrodynamic radius, determined after calcium/phosphate overload of serum samples [10]. Larger CPP2 radius is linked to higher mortality risk in dialysis patients, but this association does not appear synonymous with T50 [11]. Based on the observations of these novel indicative diagnostic approaches, mineral stress is emerging as a potentially decisive factor associated with the outcome of CKD patients [12]. Various concepts such as magnesium or spironolactone supplementation have been suggested to improve mineral stress in vulnerable populations [13, 14]. However, magnesium supplementation failed to improve vascular calcification in CKD patients [15], and further therapeutic concepts to impact on mineral stress are urgently needed.

One factor of interest is zinc, which is reduced in the serum of CKD patients [16]. Zinc deficiency has been linked to cardiovascular risk [17] and low zinc levels were recently found to be correlated with higher coronary artery calcification and cardiovascular events in CKD patients [18]. In pre-clinical experiments, zinc has been shown to mediate anti-calcific effects in vascular smooth muscle cells [19–21] and valvular interstitial cells [22]. Zinc is able to ameliorate cellular pro-calcific pathways directly via the G-protein coupled receptor 39 [20, 22]. But pilot studies also suggest a possible direct association of zinc and mineral stress in patients with CKD [20] and diabetes mellitus [23]. We therefore investigated a large cohort of blood donors and patients with renal disease with and without dialysis to substantiate a possible link of zinc and mineral stress markers in CKD.

## MATERIALS AND METHODS

### Study cohort

We conducted an explorative cross-sectional study at the Kepler University Hospital Linz, Ordensklinikum Linz and Red Cross Transfusion Service of Upper Austria in Linz, Austria. All participants provided informed written consent and approval was obtained from the local ethics commission. Patients with CKD and haemodialysis patients were recruited from 2021 to 2024 at local departments of internal medicine and nephrology based on their medical history. Inclusion criteria was known history of renal disease or dialysis treatment; patients <18 years or patients with known acute infections were excluded. Medical history and current medications were documented from clinical databases. As controls, blood donors at the local blood donation center (Red Cross Transfusion Service of Upper Austria) were recruited and medical history was collected from a questionnaire. Blood chemistry data were documented from routine clinical blood laboratory measurements where available. Urine chemistry measurements were documented from routine clinical laboratory measurements where available. Estimated glomerular filtration rate (eGFR) was calculated by the CKD Epidemiology Collaboration 2021 formula [24]. History of cardiovascular disease was defined as known coronary heart disease, peripheral artery disease, previous stroke or myocardial infarction. History of diabetes was defined as known diagnosis of diabetes mellitus.

### Functional measurements

Blood pressure data for the whole cohort were obtained from medical records or measured at the time of study inclusion. Functional measurements were performed in a subgroup of blood donors and patients with kidney disease. Pulse-wave velocity and total arterial compliance index was measured by impedance cardiography (Cardioscreen 2000, Medis) and analysed with CardiovascularLab software (Medis) [25]. In addition, pulse-wave analysis to determine augmentation index at HR 75 BPM was conducted (Sphygmocor Xcel, Atcor).

### Sample collection

Blood was collected and immediately refrigerated until serum was obtained by centrifugation. Samples were then aliquoted and stored at –80°C until further measurements. Where possible, spot urine was collected at the same time as blood collection from CKD patients and blood donors. After centrifugation, urine was aliquoted and stored at –80°C.

### Serum calcification propensity and CPP2 size

Serum calcification propensity was determined as half-maximal transformation time (T50) of primary to CPP2 as described previously [8]. Values above the measurement maximum of 570 min were set to 570 min. CPP2 hydrodynamic radius was measured by 3D-dynamic light scattering utilizing the method as described [10, 26]. Two CPP2 size measurements were excluded due to suspected aggregation during the test (values >500 nm). All measurements were performed on serum samples without freeze–thaw cycles by the reference laboratory Calciscon (Switzerland). For spiking experiments, ZnCl_2_ was added to thawed serum samples from volunteers without known diseases at 0, 10, 100 and 1000 µM concentrations. Samples were then frozen at –80°C until measurement of T50 and hydrodynamic CPP2 radius.

### Zinc measurements

Zinc concentrations in serum and urine were determined by inductively coupled plasma mass spectrometry, four serum samples could not be measured. To estimate renal excretion, urinary zinc/creatinine ratio was calculated. Furthermore, fractional excretion was calculated as (urinary zinc × plasma creatinine)/(plasma zinc × urinary creatinine) × 100 [27].

### Statistics

Data from patients are shown as median and 25th–75th percentile unless otherwise indicated. Group comparisons were performed by Kruskal–Wallis test with Dunn test or Mann–Whitney test, where appropriate. For zinc spiking experiments, Dunnetts test vs control (CTR) was used. For correlations, Spearman test was performed. For further investigations, evaluating whether zinc has effects over and above known influencing parameters, a hierarchical linear regression model was used, where T50, CPP2 size or cardiovascular parameters were defined as the dependent variable. In Step 1 of the regression model, age, sex, calcium, phosphate, magnesium and albumin were included as variables. In Step 2, zinc was additionally added. *P*-values of <.05 were considered statistically significant.

## RESULTS

### Patient characteristics

**Figure 1:**
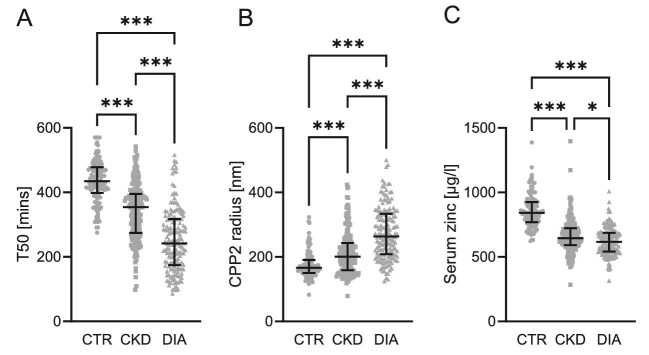
Scatter dot plots showing median and interquartile range of (**A**) serum calcification propensity (T50, min, *n* = 477), (**B**) hydrodynamic radius of CPP (CPP2, nm, *n* = 475) and (**C**) serum zinc concentrations (µg/l, *n* = 473) in blood donors (CTR), patients with chronic kidney disease (CKD) and dialysis patients (DIA). *P < .05, ^***^P < .001.

Table 1 summarizes the clinical characteristics of the recruited population. Due to limitations in the recruitment of healthy blood donors at the local blood donation centre, patients with CKD or dialysis were slightly older and included fewer female patients as compared with controls. The cohort reflected the typical alterations expected of a CKD population including hyperphosphatemia, hypocalcaemia and anaemia. T50 was lower (indicating higher serum calcification propensity) in CKD patients and further reduced in dialysis patients (Fig. 1, Table 1). The hydrodynamic radius of CPP2 increased in patients with CKD and further increased in the dialysis patients (Fig. 1, Table 1). In accordance with previous observations, serum zinc concentrations decreased in patients with renal disease. Zinc concentrations were even further reduced in dialysis patients (Fig. 1, Table 1).

**Table 1:** Descriptive characteristics of the groups in the study cohort shown as *n* number for blood donors (CTR), patients with chronic kidney disease (CKD) or dialysis patients (DIA).

	CTR	CKD	DIA	*n*/group
Female	56	87	55	120/195/162
Known CV disease	0	76	134	120/195/162
Diabetes	0	65	51	120/195/162
Calcium Pi binder	0	0	16	120/195/162
Non-calcium Pi binder	0	12	137	120/195/162
Vitamin D or analogues	1	128	122	120/195/162
Calcimimetics	0	7	62	120/195/162
Magnesium	0	12	68	120/195/162
Vitamin K antagonists	0	13	14	120/195/162
EPO/analogues	0	35	114	120/195/162
T50 (min)	435 (398–478)	354 (274–395)^***^	242 (174–317)^***,†††^	120/195/162
CPP2 radius (nm)	166 (150–191)	201 (159–243)^***^	263 (208–334)^***,†††^	119/195/161
Zinc (µg/l)	842 (769–925)	645 (592–723)^***^	617 (542–687)^***,†^	119/193/161
Age (years)	54 (47–58)	69 (58–77)^***^	64 (54–75)^***^	120/195/162
eGFR (ml/min/1.73 m²)	93.5 (85.2–100.3)	23.3 (13.5–34.4)^***^	n.d.	120/195/0
Calcium (mmol)	2.38 (2.30–2.44)	2.32 (2.25–2.40)^***^	2.18 (2.08–2.28)^***,†††^	119/194/162
Phosphate (mmol)	0.95 (0.83–1.04)	1.23 (1.05–1.47)^***^	1.86 (1.50–2.19)^***,†††^	120/195/162
Magnesium (mmol)	0.86 (0.82–0.89)	0.87 (0.78–0.95)	1.00 (0.90–1.10)^***,†††^	120/195/162
BUN (mg/dl)	14 (11–16)	44 (29–62)^***^	58 (48–66)^***,†††^	120/195/162
Albumin (mg/dl)	4640 (4443–4798)	4250 (4070–4450)^***^	4075 (3858–4290)^***,†††^	120/195/162
Hb (g/dl)	14.7 (13.7–15.5)	12.2 (11.2–13.5)^***^	11.5 (10.4–12.1)^***,†††^	120/194/161
Systolic RR (mmHg)	138 (127–149)	150 (139–168)^***^	142 (126–159)^†††^	120/169/159
Diastolic RR (mmHg)	87 (79–96)	84 (77–92)	75 (66–85)^***,†††^	120/169/159
Pulse pressure (mmHg)	53 (43–61)	67 (56–80)^***^	66 (54–80)^***^	120/169/159
PWV (m/s)	6.8 (6.2–7.4)	8.4 (7.1–9.6)^***^	n.d.	53/161/0
TACI (ml/m²/mmHg)	0.98 (0.83–1.18)	0.68 (0.55–0.85)^***^	n.d.	53/154/0
Aortic AIX (AP/PP) HR75 (%)	29 (21–39)	31 (19–39)	n.d.	30/118/0

Median and interquartile range for age, eGFR and blood chemistry as well as blood pressure and further vascular measurements of the groups in the study cohort.

^***^ *P* < .001 vs CTR; ^†^*P* < .05, ^†††^*P* < .001 vs CKD.

CV, cardiovascular; Pi, phosphate; EPO, erythropoietin; BUN, blood urea nitrogen; Hb, haemoglobin; RR, blood pressure, PWV, pulse-wave velocity.

### Association of zinc with T50 and CPP2 radius

**Figure 2:**
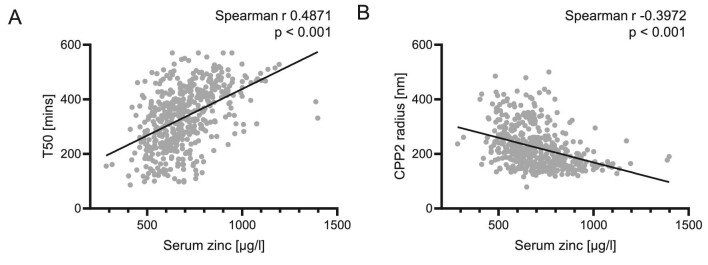
Correlations of serum zinc with (**A**; *n* = 473) serum calcification propensity (T50) and (**B**; *n* = 471) hydrodynamic radius of CCP (CPP2) in the whole-study cohort. *P*-values are indicated in the figure.

Next, the associations of the mineral stress markers T50 and CPP2 radius were investigated. In this study cohort, serum zinc levels were positively correlated to T50 (Fig. 2A). When stratifying by groups, T50 was significantly correlated to serum zinc levels in CKD patients (Spearman r 0.2469, *P *= .001, *n* = 193), but this correlation did not reach statistical significance in blood donors (Spearman r 0.1541, *P *= .094, *n* = 119) or dialysis patients alone (Spearman r 0.1018, *P *= .199, *n* = 161). Investigating the CKD and dialysis groups together, the correlation of zinc and T50 remained significant (Spearman r 0.2468, *P *< .001, *n* = 354).

Based on these observations, further analysis was performed to determine whether the associations of serum zinc with mineral stress parameters were independent of other known factors and explained additional variance when the influences of these known factors are already taken into account. To this end, a hierarchical linear regression model was conducted with T50 as dependent variable (Table 2). The first step of the model (F *P *< .001) included age, sex as well as calcium, phosphate, magnesium and albumin—based on the rationale that these are main factors driving serum calcification propensity in renal disease and no valid measurement of residual renal function was available in this population including dialysis patients. Except sex, all variables were significantly associated with T50 in this cohort. In Step 2, zinc was included, resulting in an improved prediction of the model (significant change in F: *P *= .007), confirming the additional contribution of zinc to the expression of T50. Serum zinc concentrations showed therefore a significant association with T50.

**Table 2:** Factors associated with serum calcification propensity T50 (*n* = 472) in the study cohort in a hierarchical regression model [dependent variable T50, Model 1 includes age, sex, calcium, phosphate, magnesium and albumin (change in F: *P *< .001), Model 2 further includes zinc (change in F: *P *= .007)], shown with regression coefficients (beta) and levels of significance (*P*-value; bolded if *P* < .05).

	Model 1		Model 2	
T50 (min)	Beta	*P*	Beta	*P*
Age (years)	**–0.117**	**.000**	**–0.101**	**.001**
Sex (m1/f0)	–0.008	.766	–0.008	.777
Calcium (mmol)	**0.101**	**.002**	**0.096**	**.004**
Phosphate (mmol)	**–0.713**	**.000**	**–0.691**	**.000**
Magnesium (mmol)	**0.297**	**.000**	**0.292**	**.000**
Albumin (mg/dl)	**0.178**	**.000**	**0.145**	**.000**
Zinc (µg/l)			**0.090**	**.007**

Furthermore, lower serum zinc levels were correlated to a higher hydrodynamic radius of CPP2 (Fig. 2B). Stratified by groups, a significant correlation for CPP2 radius and serum zinc was observed in CKD patients (Spearman r –0.1931, *P *= .007, *n* = 193) and in dialysis patients (Spearman r –0.1559, *P *= .049, *n* = 160), but not in blood donors (Spearman r –0.1116, *P *= .229, *n* = 118). Investigating the CKD and dialysis groups together, the correlation of zinc and CPP2 radius remained significant (Spearman r –0.2234, *P *< .001, *n* = 353). A similar hierarchical linear regression model was conducted with CPP2 radius as dependent variable (Table 3). Step 1 (F *P *< .001) again included age, sex as well as calcium, phosphate, magnesium and albumin, and all except sex were significantly associated with CPP2 radius. Adding zinc to the model in Step 2 resulted in a significant change in F (*P *= .010) and zinc was significantly associated with CPP2 radius, while albumin did not reach statistical significance.

**Table 3:** Factors associated with hydrodynamic radius of CPP2 (*n* = 470) in the study cohort in a hierarchical regression model [dependent variable CPP2 radius, Model 1 includes age, sex, calcium, phosphate, magnesium and albumin (change in F: *P *< .001), Model 2 further includes zinc (change in F: *P *= .010)], shown with regression coefficients (beta) and levels of significance (*P*-value; bolded if *P* < .05).

	Model 1		Model 2	
CPP2 radius (nm)	Beta	*P*	Beta	*P*
Age (years)	**0.208**	**.000**	**0.186**	**.000**
Sex (m1/f0)	0.053	.180	0.052	.183
Calcium (mmol)	**–0.177**	**.000**	**–0.171**	**.000**
Phosphate (mmol)	**0.237**	**.000**	**0.206**	**.000**
Magnesium (mmol)	**0.129**	**.003**	**0.136**	**.002**
Albumin (mg/dl)	**–0.118**	**.015**	–0.073	.152
Zinc (µg/l)			**–0.125**	**.010**

**Figure 3:**
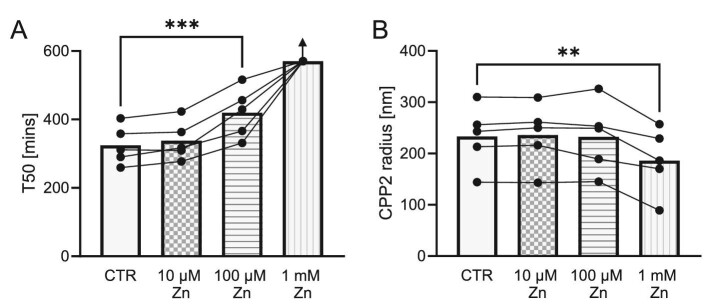
T50 (**A**) and hydrodynamic radius of CPP2 (**B**) shown as mean (bars) with individual measurements depicted as dots in serum samples from healthy volunteers (*n* = 5) after addition of ZnCl_2_ (0–1000 µM). All T50 values for the 1 mM zinc group were above the measurement range of 570 min (indicated by arrow), and therefore excluded from statistical analysis (Dunnetts test vs CTR). ^**^P < .01, ^***^P < .001.

To indicate a putative direct effect of zinc on CPP formation, we added various concentrations of zinc or vehicle to serum samples of healthy volunteers (Fig. 3). Since the T50 test is conducted with supraphysiological levels of calcium and phosphate, we added a physiological concentration (10 µM corresponding to 653.8 µg/l) and also a larger concentration range of higher concentrations. Addition of 100 µM zinc (corresponding to 6538 µg/l) significantly prolonged T50, and addition of 1 mM zinc elevated T50 beyond the measurement range of 570 min in all samples. However, the hydrodynamic radius of CPP2 was significantly reduced only in samples with 1 mM zinc added.

### Association of zinc with cardiovascular parameters

**Figure 4:**
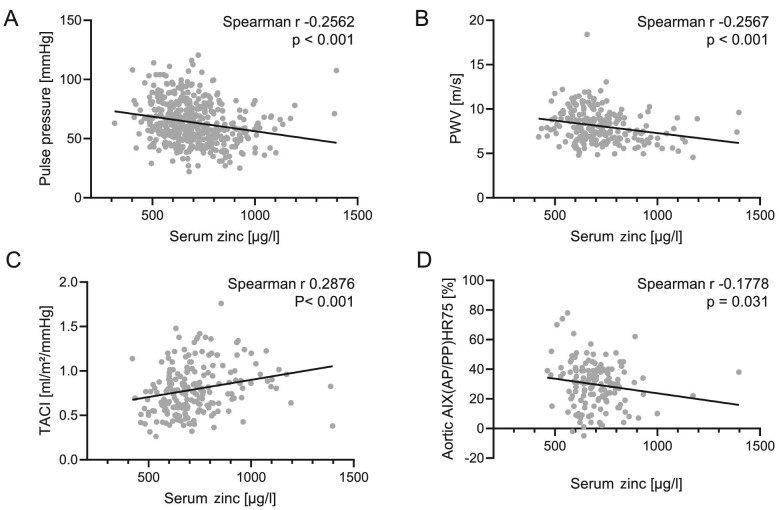
Correlations of serum zinc with pulse pressure (**A**; *n* = 446) in the whole cohort, as well as pulse-wave velocity (PWV, **B**; *n* = 213), total arterial compliance index TACI (**C**; *n* = 206) and aortic augmentation index at heart rate 75 (AIX, **D**; *n* = 148) in blood donors and CKD patients. *P*-values are indicated in the figure.

Next, we investigated whether serum zinc concentrations were linked to cardiovascular functional parameters (Fig. 4). Serum zinc was inversely correlated to pulse pressure, where data was available from most of the study cohort. To study vascular functional alterations, we investigated pulse-wave velocity, total arterial compliance index (TACI) and aortic augmentation index at HR75 in a subgroup of blood donors and CKD patients, as these parameters apparently cannot feasibly be obtained in dialysis patients due to large fluctuations of measurements [28]. A negative correlation of serum zinc was observed with pulse-wave velocity and aortic augmentation index at HR75. Total arterial compliance index showed a positive correlation with serum zinc levels. However, when utilizing the hierarchical linear regression model, no significant association with zinc was observed (Table 4). Pulse pressure showed a significant F when the Model 1 factors were included (Step 1; *P *< .001), showing age, phosphate and albumin as significant determinants. But addition of zinc in Step 2 of the model did not improve the model, as no significant change in F for zinc (*P *= .683) was observed. Also, for pulse-wave velocity the model was significant (Step 1; F *P *< .001) with significant associations for age, sex, phosphate and magnesium, but adding zinc in Step 2 did not result in significant change in F (*P *= .813). Similar observations were observed for TACI (F *P *< .001; Step 2 change in F: *P *= .405) and aortic augmentation index HR75 (F *P *= .001; Step 2 change in F: *P *= .237).

**Table 4:** Factors associated with pulse pressure (*n* = 446) in the whole cohort as well as pulse-wave velocity (PWV, *n* = 213), TACI (*n* = 206) and aortic augmentation index HR75 (AIX, *n* = 148) in blood donors and patients with CKD in a hierarchical regression model (dependent variables: pulse pressure, PWV, TACI or AIX, Model 1 includes age, sex, calcium, phosphate, magnesium and albumin, Model 2 further includes zinc), shown with regression coefficients (beta) and levels of significance (*P*-value; bolded if *P* < .05).

	Model 1		Model 2	
	Beta	*P*	Beta	*P*
Pulse pressure (mmHg)				
Age (years)	**0.327**	**.000**	**0.330**	**.000**
Sex (m1/f0)	0.003	.940	0.003	.941
Calcium (mmol)	0.046	.387	0.045	.396
Phosphate (mmol)	**0.192**	**.000**	**0.198**	**.000**
Magnesium (mmol)	–0.010	.823	–0.011	.817
Albumin (mg/dl)	**–0.129**	**.016**	**–0.138**	**.017**
Zinc (µg/l)			0.022	.683
PWV (m/s)				
Age (years)	**0.556**	**.000**	**0.553**	**.000**
Sex (m1/f0)	**0.199**	**.000**	**0.199**	**.000**
Calcium (mmol)	0.098	.131	0.097	.133
Phosphate (mmol)	**0.150**	**.018**	**0.146**	**.029**
Magnesium (mmol)	**–0.151**	**.008**	**–0.150**	**.009**
Albumin (mg/dl)	–0.090	.177	–0.085	.220
Zinc (µg/l)			–0.015	.813
TACI (ml/m²/mmHg)				
Age (years)	**–0.597**	**.000**	**–0.609**	**.000**
Sex (m1/f0)	–0.039	.479	–0.037	.498
Calcium (mmol)	–0.038	.555	–0.040	.541
Phosphate (mmol)	**–0.201**	**.002**	**–0.216**	**.001**
Magnesium (mmol)	0.022	.699	0.024	.675
Albumin (mg/dl)	0.045	.501	0.062	.378
Zinc (µg/l)			–0.055	.405
Aortic AIX HR75 (%)				
Age (years)	**0.217**	**.008**	**0.202**	**.014**
Sex (m1/f0)	**–0.283**	**.000**	**–0.286**	**.000**
Calcium (mmol)	–0.085	.362	–0.082	.381
Phosphate (mmol)	0.066	.492	0.044	.654
Magnesium (mmol)	–0.041	.643	–0.034	.702
Albumin (mg/dl)	–0.010	.918	0.021	.832
Zinc (µg/l)			–0.105	.237

### Urinary zinc, mineral stress markers and renal function

We further investigated renal excretion of zinc in a subgroup with available urine samples (Table 5) and estimated zinc excretion by calculating the urinary zinc to creatinine ratio (µg/mg) and fractional excretion (FE) of zinc. As a result, zinc excretion was increased in patients with CKD. Furthermore, we investigated an association of zinc excretion and mineral stress markers. Zinc FE was correlated with serum calcification propensity and CPP2 diameter (Supplementary data, Fig. S1). However, in the hierarchical linear regression model (Supplementary data, Table S1), addition of FE zinc in Step 2 did not show a significant effect for T50 (change in F: *P *= .122) or CPP2 diameter (change in F: *P *= .321). Similarly, FE zinc was positively correlated with pulse pressure and pulse-wave velocity, and negatively with TACI (Supplementary data, Fig. S2). No correlation was found for FE zinc and aortic augmentation index HR75. Again, utilizing the hierarchical linear regression model (Supplementary data, Table S2), addition of FE zinc did not show a significant effect for pulse pressure (change in F: *P *= .318), pulse-wave velocity (change in F: *P *= .106) or aortic augmentation index (change in F: *P *= .449). However, a significant effect of FE zinc on total arterial compliance index was observed (change in F: *P *= .045).

**Table 5:** Zinc excretion as estimated from zinc to creatinine ratio or calculated FE in blood donors (CTR, *n* = 49) and patients with chronic kidney disease (CKD, *n* = 184).

	CTR	CKD
Urinary zinc to creatinine ratio (µg/mg)	0.312 (0.223–0.447)	0.422 (0.276–0.631)^**^
FE zinc (%)	0.32 (0.22–0.47)	1.77 (1.18–2.60)^***^

^**^ *P *< .01, ^***^P < .001.

Next, we also investigated the influence of renal function on zinc homeostasis in non-dialysis CKD patients and blood donors. In CKD patients, serum zinc concentrations were not significantly correlated to eGFR (Spearman r 0.0902, *P *= .212, *n* = 193). In contrast, higher fractional zinc excretion was significantly correlated to lower eGFR (Spearman r –0.4780, *P *< .001, *n* = 184). In blood donors, higher serum zinc concentrations were correlated with higher eGFR (Spearman r 0.2732, *P *= .003, *n* = 119). As opposed to CKD patients, no correlation of fractional zinc excretion and eGFR was observed in blood donors (Spearman r –0.0144, *P *= .922, *n* = 49).

## DISCUSSION

This is the largest study to our knowledge on zinc as an independent determinant of mineral stress indicators in patients with reduced renal function. As expected, zinc levels declined in patients with CKD. This is in accordance with previous studies as concluded by meta-analyses [16, 29]. In our cohort, dialysis patients exhibited even slightly lower zinc levels than non-dialysis CKD patients, a difference not observed in a previous meta-analysis [16]. However, data on the comparison between pre-dialysis CKD patients and dialysis CKD patients are sparse, based on low sample sizes and biased by large differences in publication year [16]. Nonetheless, the effect of dialysis on serum zinc appears rather complex. Although reduced serum zinc concentrations are consistently shown in dialysis patients versus controls, zinc levels are higher in blood after a dialysis session compared with before [30]. Thus, a loss of zinc in the dialysate does not appear as a primary major cause for the reduced serum zinc in dialysis patients. Further studies are required to discriminate the effects of dialysis versus pre-dialysis CKD and to identify underlying causes of zinc deficiency. Due to the monocentric approach, our observations of zinc levels in CKD versus dialysis patients should be interpreted with caution, as differences in dialysis regimen or nutritional habits might complicate generalization of the results.

Zinc deficiency in patients with CKD has been linked to a combined effect of increased urinary loss [31] and insufficient intestinal uptake [32]. Although we measured only spot urine samples, an increased zinc excretion in CKD patients was indicated despite reduced serum zinc concentrations, confirming previous observations [31]. Contrary to blood donors, zinc excretion but not serum zinc levels were correlated with eGFR in CKD patients. This indicates that urinary zinc loss could be a marker for renal function in CKD. In turn, zinc deficiency has been suggested as a risk factor for CKD progression [33]. An impaired tubular function is suspected to cause increased urinary zinc excretion in CKD, but the underlying mechanisms are unclear [31].

The most important finding of this study is an association of serum zinc concentrations with T50. As our cohort included dialysis patients, no valid marker of renal function is available. Thus, we included the main determinants how GFR alters T50 in our analysis as discussed previously [34]. In this relevant model, the association of zinc remained even when including relevant factors such as age, sex and phosphate, indicating an independent effect. In contrast, the correlation of urinary zinc excretion with T50 and CPP2 radius was not independent of other factors, but this analysis is based on a lower sample size. Although a significant association of zinc and hydrodynamic radius of CPP2 was observed, the zinc-spiking experiments over a large concentration range indicate, that the direct effect of zinc may stronger affect T50 as compared with CPP2 radius. CPP2 radius appeared unchanged at high zinc concentrations which were already able to increase T50. However, clearly a small-scale pilot experiment cannot rule out a direct effect of zinc on CPP2 radius. Also, the *ex vivo* situation of serum with supraphysiological zinc, calcium and phosphate levels must be interpreted with caution.

T50 has emerged as an important factor linked to mortality of CKD patients [7]. Besides CKD patients [35, 36], investigations of T50 extended to other patient collectives such as the general population [9], pseudoxanthoma elasticum [37], aldosteronism [38], systemic lupus erythematodes [39] and ischaemic heart failure [40]. The data on the role of CPP2 radius are more sparse, but it was shown to correlate with mortality risk in dialysis patients [11] and peripheral artery disease patients [26]. It is therefore tempting to speculate that zinc supplementation could improve T50 and thereby reduce mineral stress in CKD. Zinc is an essential nutrient and malnutrition is common in CKD [41]. In haemodialysis patients, inadequate zinc intake is highly prevalent [42]. Reduced dietary zinc intake may increase the risk of CKD [43] and vascular calcification [44]. Several studies investigating zinc supplementation in dialysis patients were reported [45]. Thus, improvement of T50 by zinc supplementation seems a feasible therapeutic concept for further investigation. However, this must be carefully considered, since zinc supplementation may be associated with side effects, such as impaired copper absorption in the intestine and favouring copper deficiency [46].

Zinc was further correlated to indicators of arterial stiffness as determined by pulse-wave velocity, augmentation index HR75 and total arterial compliance index, but these associations were not significant in a multivariate model. Only urinary zinc excretion showed a significant association with total arterial compliance index, a predictor of mortality in the general population [47]. However, our sample size in these measurements was small and we excluded dialysis patients, since dialysis patients show cyclic change in pulse-wave velocity with wide variations [48]. Nonetheless, an association of serum zinc and pulse-wave velocity was recently suggested in dialysis patients [49]. But the current study design may not be ideally suited to investigate this interaction. This study included a single timepoint of T50 and zinc measurement. These serum parameters might be rather dynamic and exhibit short-term fluctuations due to dietary status [50, 51], seasonal variations [52] or other factors. Measurements of vascular stiffness such as pulse-wave velocity could be considered indicators structural alterations due to long-term remodelling processes [53]. Thus, a single blood sample might be suited to associate T50 with serum zinc, but may be an insufficient representation of chronic zinc status, where acute fluctuation might mask the association with vascular stiffness. Therefore, the current findings cannot rule out a link between zinc and vascular stiffness.

The cardiovascular effects of zinc are most likely not solely mediated by a direct regulation of T50. Zinc may alter cardiomyocyte function [54]. Anti-calcific effects of zinc are described in vascular smooth muscle cells [21]. The inhibition of cellular calcification by zinc is partly mediated through the zinc-sensing receptor GPR39 [19, 20, 22]. Activation of this receptor has a complex effect on inflammatory pathways [20] and inhibition of nuclear factor-κB might be an important mediator for its anti-calcific effects [55], but a status of increased inflammation might also impair T50 [56] and increase pulse-wave velocity [57]. Ultimately, further studies are required to elucidate the link between zinc, mineral stress and vascular alterations.

Our study has several strengths and limitations. This is to our knowledge the largest study linking zinc in renal disease to mineral stress markers and includes a wide range of renal function from blood donors to dialysis patients. The uniform processing of samples ensures comparability of mineral stress measurements at a single timepoint together with zinc measurements. However, the monocentric approach is prone to bias, and the limitations in recruitment of blood donors resulted in a small, but significant age difference between blood donors and patients with renal disease, a problem also previously encountered in similar approaches [31]. Also, we included blood donors/CKD patients based on their suitability as blood donor or medical history of renal disease, therefore this approach cannot provide a sharp distinction of healthy versus CKD. Furthermore, the data from this cohort originate from several recruitment sites and lack other relevant biomarkers in CKD, such as iron homeostasis, vitamin D levels, parathyroid hormone or alkaline phosphatase. It is incompletely clarified to what extent medications and various phosphate binders may affect zinc metabolism [58–60]. Unknown or unmeasured determinants not included in our multivariate model might alter the observed associations. A 24 h urine collection was not possible in this approach, so the current data rely on spot urine samples and estimated urine calculations. Also, we are lacking nutritional data on zinc intake or gastrointestinal loss and we could not obtain fasting serum samples. Individual nutritional status might introduce some variation, which could especially mask associations of serum measurements with functional data. Data derived from impedance cardiography might itself be limited in this setting [61]. Due to the design of the approach, we had to rely on cross sectional data without multiple measurements, and causality cannot be interpreted.

In conclusion, this study shows an independent association of low serum zinc concentrations with increased mineral stress markers in patients with CKD. A possible functional role of zinc in vascular stiffening requires further study. Further long-term and interventional studies with zinc supplementation may open a new therapeutic avenue to counter mineral stress in patients with renal disease.

## Supplementary Material

sfae258_Supplemental_File

## Data Availability

The data underlying this article will be shared on reasonable request to the corresponding author.
