# Supplementary material for: Association of serum zinc with mineral stress in chronic kidney disease
Source: Clin Kidney J. 2024 Aug 20;17(9):sfae258. doi: 10.1093/ckj/sfae258 (PMC11403325; doi:10.1093/ckj/sfae258)
Supplement: sfae258_Supplemental_File [file sfae258_supplemental_file.pdf]

Supplementary material

Association of serum zinc with mineral stress in chronic kidney disease

Suppl. Figure 1. Correlations of fractional excretion zinc (FE zinc, %) with (**A**, n=233) serum calcification propensity (T50) and (**B**, n=232) hydrodynamic radius of calciprotein particles (CPP2) in blood donors and CKD patients. P values are indicated in the figure.

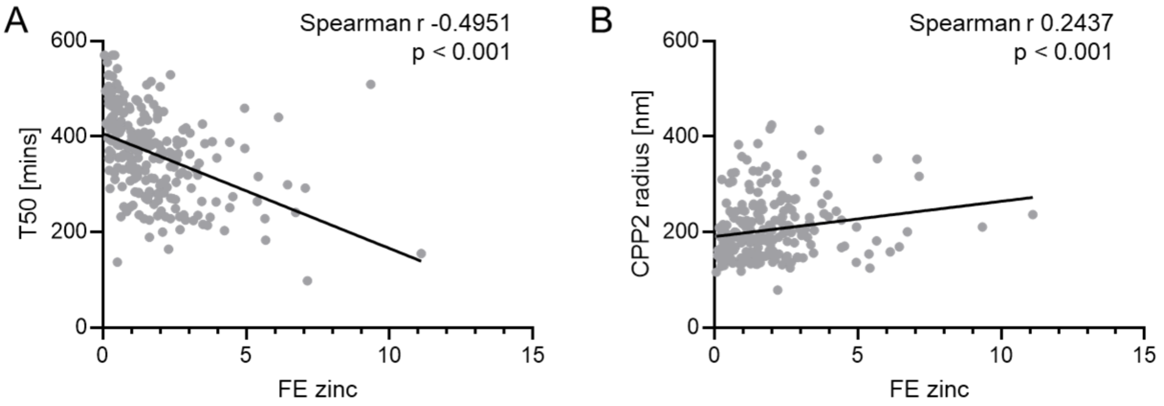

Suppl. Figure 2. Correlations of fractional excretion zinc (FE zinc, %) with pulse pressure (**A**; n= 210), as well as pulse wave velocity (PWV, **B**; n=202), total arterial compliance index TACI (**C**; n=195) and aortic augmentation index at heart rate 75 (AIX, **D**; n=142) in blood donors and CKD patients. P values are indicated in the figure.

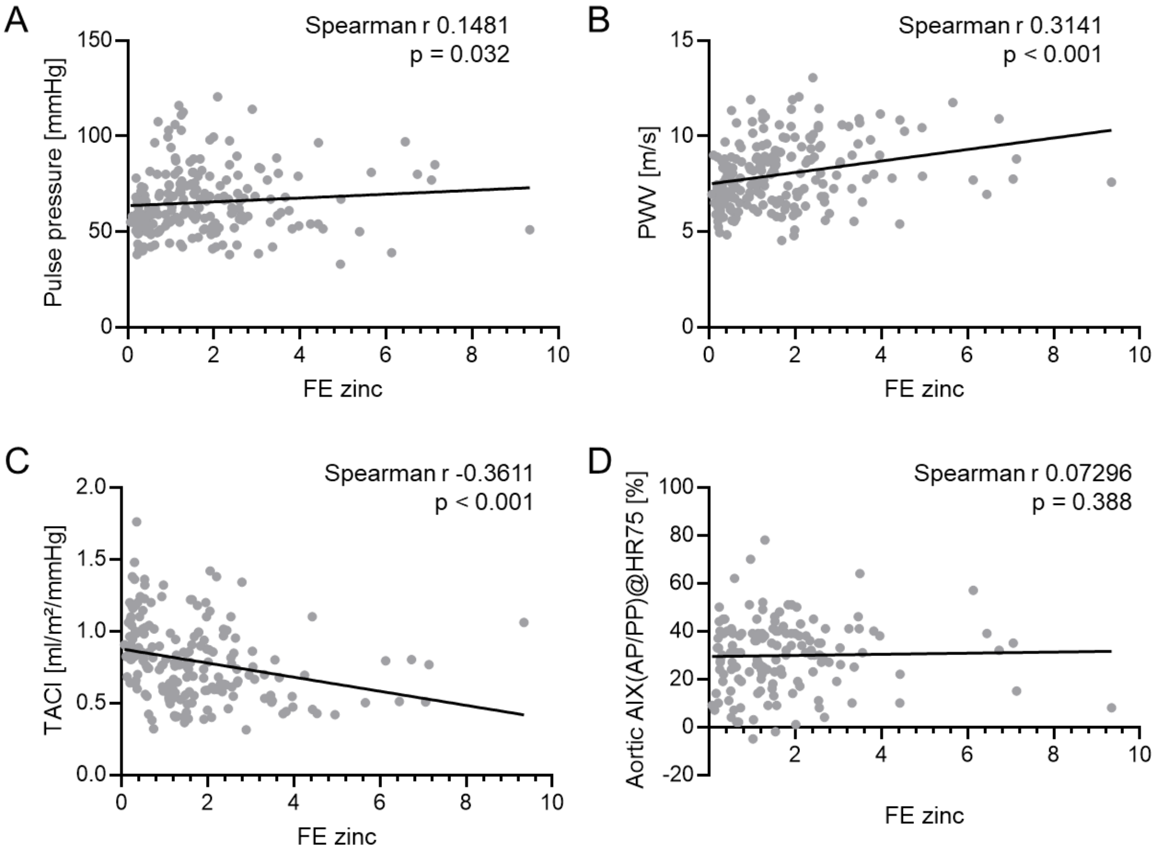

Suppl. Table 1. Factors associated with serum calcification propensity (T50, n=232) and hydrodynamic radius of CPP2 (n=231) in blood donors and patients with renal disease in a hierarchical regression model (dependent variable T50 or CPP2 radius, model 1 includes age, sex, calcium, phosphate, magnesium and albumin, model 2 further includes fractional excretion of zinc), shown with regression coefficients (beta) and levels of significance (p value; bolded if $p < 0.05$).

T50 (mins)	Model 1		Model 2	
	beta	p	beta	p
Age (yrs)	**-0.097**	**.032**	**-0.091**	**.045**
Sex (m1/f0)	0.025	.563	0.039	.383
Calcium (mmol)	**0.134**	**.010**	**0.145**	**.006**
Phosphate (mmol)	**-0.587**	**.000**	**-0.545**	**.000**
Magnesium (mmol)	**0.418**	**.000**	**0.407**	**.000**
Albumin (mg/dl)	0.024	.650	0.005	.927
FE zinc (%)			-0.083	.122

CPP2 radius (nm)	Model 1			
	beta	p	beta	p
Age (yrs)	**0.291**	**.000**	**0.286**	**.000**
Sex (m1/f0)	0.016	.797	0.003	.955
Calcium (mmol)	**-0.185**	**.011**	**-0.195**	**.008**
Phosphate (mmol)	**0.157**	**.027**	0.119	.138
Magnesium (mmol)	**0.139**	**.029**	**0.149**	**.021**
Albumin (mg/dl)	-0.015	.833	0.002	.984
FE zinc (%)			0.075	.321

Suppl. Table 2. Factors associated with pulse pressure (n=210), pulse wave velocity (n=202), TACI (n=195) and aortic augmentation index HR 75 (n=142) in blood donors and patients with renal disease in hierarchical regression models (dependent variables: pulse pressure, PWV, TACI or AIX, model 1 includes age, sex, calcium, phosphate, magnesium and albumin, model 2 further includes fractional excretion of zinc), shown with regression coefficients (beta) and levels of significance (p value, bolded if p < 0.05).

Pulse pressure (mmHg)	Model 1		Model 2	
	beta	p	beta	p
Age (yrs)	**0.411**	**.000**	**0.416**	**.000**
Sex (m1/f0)	-0.087	.158	-0.076	.229
Calcium (mmol)	-0.057	.439	-0.048	.520
Phosphate (mmol)	0.085	.234	0.119	.132
Magnesium (mmol)	0.073	.248	0.065	.306
Albumin (mg/dl)	-0.135	.067	**-0.153**	**.044**
FE zinc (%)			-0.074	.318

PWV (m/s)	Model 1		Model 2	
	beta	p	beta	p
Age (yrs)	**0.564**	**.000**	**0.556**	**.000**
Sex (m1/f0)	**0.195**	**.001**	**0.180**	**.002**
Calcium (mmol)	0.073	.271	0.056	.399
Phosphate (mmol)	**0.185**	**.005**	0.136	.059
Magnesium (mmol)	**-0.164**	**.005**	**-0.151**	**.011**
Albumin (mg/dl)	-0.039	.566	-0.007	.922
FE zinc (%)			0.109	.106

TACI (ml/m²/mmHg)	Model 1		Model 2	
	beta	p	beta	p
Age (yrs)	**-0.580**	**.000**	**-0.571**	**.000**
Sex (m1/f0)	-0.019	.741	0.004	.948
Calcium (mmol)	-0.031	.651	-0.014	.837
Phosphate (mmol)	**-0.208**	**.002**	**-0.148**	**.045**
Magnesium (mmol)	0.022	.715	0.009	.876
Albumin (mg/dl)	0.022	.761	-0.021	.778
FE zinc (%)			**-0.140**	**.045**

Aortic AIX HR75 (%)	Model 1		Model 2	
	beta	p	beta	p
Age (yrs)	0.162	.054	0.159	.059
Sex (m1/f0)	**-0.294**	**.000**	**-0.308**	**.000**
Calcium (mmol)	-0.112	.247	-0.125	.203
Phosphate (mmol)	0.046	.640	0.012	.915
Magnesium (mmol)	-0.042	.643	-0.037	.686
Albumin (mg/dl)	0.036	.712	0.062	.547
FE zinc (%)			0.077	.449
